# Spatial Evaluation of Soil Moisture (SM), Land Surface Temperature (LST), and LST-Derived SM Indexes Dynamics during SMAPVEX12

**DOI:** 10.3390/s19051247

**Published:** 2019-03-12

**Authors:** Hao Sun, Baichi Zhou, Hongxing Liu

**Affiliations:** 1 College of Geoscience and Surveying Engineering, China University of Mining and Technology, Beijing 100083, China; 809144168zbc@gmail.com; 2 Department of Geography, University of Alabama, Tuscaloosa, AL 35487, USA; Hongxing.Liu@ua.edu

**Keywords:** soil moisture, land surface temperature, downscaling, LST/FVC space

## Abstract

Downscaling microwave soil moisture (SM) with optical/thermal remote sensing data has considerable application potential. Spatial correlations between SM and land surface temperature (LST) or LST-derived SM indexes (SMIs) are vital to the current optical/thermal and microwave fusion downscaling methods. In this study, the spatial correlations were evaluated at the same spatial scale using SMAPVEX12 SM data and MODIS day/night LST products. LST-derived SMIs was calculated using NLDAS-2 gridded meteorological data with conventional trapezoid and two-stage trapezoid models. Results indicated that (1) SM agrees better with daytime LST than the nighttime or the day-night differential LST; (2) the daytime LSTs on Aqua and Terra present very similar spatial agreement with SM and they have very similar performances as downscaling factors in simulating SM; (3) decoupling effect among SM, LST, and LST-derived SMIs occurs not only in very wet but also in very dry condition; and (4) the decoupling effect degrades the performance of LST as a downscaling factor. The future downscaling algorithms should consider net surface radiation and soil type to tackle the decoupling effect.

## 1. Introduction

Soil moisture (SM) is an important water source for land surface evapotranspiration (ET) and crop growth. It also plays a significant role in partitioning available energy into sensible heat flux and latent heat flux as well as partitioning precipitation into penetration and runoff. Therefore, it is an essential variable in many fields such as agricultural drought monitoring [1], water source management [2], and climate change [3], etc. Various in situ techniques have been developed to measure soil moisture with ground instruments such as gravimetric methods, time domain reflectometry, and cosmic-ray neutron probes [4], etc. However, these soil moisture measurements are very limited in capturing the spatial distribution of soil moisture due to the high spatial heterogeneity impacted by soil texture and structure, topographic features, land cover patterns, and meteorological forcing conditions at various scales [5].

Remote sensing technology, especially microwave remote sensing at the L-band, is a powerful tool to obtain SM from regional to global scales and at a temporal resolution of few days [3,6,7]. However, the SM data retrieved from microwave, especially passive microwave remote sensing, has coarse spatial resolution due to trade-offs between spatial, spectral, and radiometric resolution. SMOS (Soil Moisture and Ocean Salinity) [8] and SMAP (Soil Moisture Active Passive) [9] are two operational satellite missions that are based on the L-band for remote sensing soil moisture. The SMOS, launched on 2 November 2009, provides multi-angular (0°–65°) and full-polarimetric observations with a spatial resolution of ~35–50 km [10]. The SMAP, launched on 31 January 2015, carries two payloads: a real aperture radiometer and a Synthetic Aperture Radar (SAR), providing a single angle (40°) and full-polarimetric observations at spatial resolutions of ~36 km and ~3 km, respectively. SMAP planned to provide soil moisture products at low (~40 km), high (~3 km) and intermediate (~10 km) spatial resolutions using observations from radiometer, SAR, and a combination of the two, respectively. Unfortunately, the SAR portion experienced a fatal anomaly, which caused the radar to stop transmitting data on 7 July 2015 [11]. In short, the soil moisture data at a spatial resolution of dozens of kilometers is too coarse to support hydrological and agricultural applications on a regional or local scale. Spatial downscaling of microwave soil moisture to higher resolutions such as one kilometer or even hundreds of meters is required for regional applications [3,4].

Downscaling microwave soil moisture with optical/thermal remote sensing data has presented considerable potential due to its high spatial resolution, abundant data source, and relative mature observation technology, as well as the well-defined basic physics to link them with SM [4,12,13,14]. Currently, there are three categories of optical/thermal and microwave fusion methods— statistical regression, relative ratio, and physical model for simplification. Within the statistical regression method, empirical polynomial fitting [15] or machine learning algorithms [16] are used to construct a statistical relationship between soil moisture and various land surface parameters such as Land Surface Temperature (LST), Normalized Difference Vegetation Index (NDVI), Albedo [15], and microwave brightness temperature [17], etc. For relative ratio methods, high-resolution soil moisture is considered proportional to coarse-resolution soil moisture with a soil wetness index [14]. The soil wetness index is actually calculated from the feature space of LST and Fractional Vegetation Coverage or vegetation index (LST/FVC space) [18,19]. The relative ratio methods were further improved by introducing Vegetation Temperature Condition Index (VTCI) or Temperature Vegetation Dryness Index (TVDI) [20,21]. With regard to the physical model, Disaggregation based on Physical And Theoretical scale CHange (DISPATCH) developed by Merlin et al. is a representative [22,23]. Its physical basis is the linear, exponential, or cosine expressions between soil moisture and soil evaporative efficiency (SEE). SEE is defined as the ratio of actual soil evaporation to the potential one. In the DISPATCH model, SEE is calculated as the relative distance of soil temperature to its extreme values at the conditions of maximum water stress and saturated water supply [23]. The soil temperature and its extreme values were actually determined from the LST/FVC space. In view of this, the determination of SEE is very similar to the calculation of VTCI or TVDI, which are all derived from the LST/FVC space. We call them the LST-derived soil moisture indices (SMIs).

The LST and LST-derived SMIs are vital to the optical/thermal and microwave fusion methods for disaggregating coarse-resolution SM data into a higher resolution. Therefore, gaining an understanding of the LST-SM link and LST derived SMI-SM link from observations could help refine the disaggregation of coarse-resolution SM [10]. It has been known that SM exerts a complicated influence on LST in three different ways. Firstly, SM has a positive correlation with surface thermal inertia. Thus, a decrease in SM generally leads to a decrease in thermal inertia and an increase in LST diurnal range. The daily maximum LST over the wet surface appears later than that over the dry surface. Secondly, SM has positive influences on ET within a specific range of SM from permanent wilting point ($\theta_{\mathrm{WILT}}$) to a given critical SM value ($\theta_{\mathrm{CRIT}}$) [24]. Within this range, SM provides a first-order constraint on ET (called SM-limited ET regime) and thus an increase in SM produces a decrease in LST. Above the $\theta_{\mathrm{CRIT}}$, ET rate is independent of the SM (called energy-limited ET regime). Under the energy-limited ET regime, SM has insignificant correlation with LST. Thirdly, soil emissivity increases with the increase of SM. Systematic errors from 0.1 K to 2 K can be caused by SM influence on emissivity in retrieving LST [25]. Pablos et al. [10,26] conducted temporal correlation analysis between SM and LST using in situ and made remote sensing observations. Results from in situ data show that instantaneous SM exhibits stronger anti-correlation to daily maximum LST than to instantaneous LST, daily mean LST, daily median LST, daily minimum LST and LST diurnal range. When comparing SMOS SM with MODIS (or Moderate Resolution Imaging Spectroradiometer) LST from Terra/Aqua day/night, stronger anti-correlation is obtained between SMOS SM and MODIS LST day than night in both Terra and Aqua platforms [10,26]. However, the correlation analysis between SM and LST is mainly focusing on the temporal scale and using SM and LST with different spatial resolutions (i.e., ~25 km for SMOS SM and ~1 km for MODIS LST). Note that many methods of downscaling coarse-resolution SM take advantage of the spatial variation of LST in depicting SM variation [15,17]. Particularly, the min-max normalized LST rather than the LST itself was used to construct polynomial regression formula with SM [15,17,27]. Therefore, in addition to the temporal dynamics, evaluating spatial dynamic of SM and LST at the same spatial resolution is also essential for understanding the LST-SM link. Furthermore, many methods utilize LST-derived SMI to disaggregate coarse-resolution SM [4]. Thus, the spatial dynamic of SM and LST-derived SMI at the same spatial scale is also worthy of further investigation in order to refine the SM disaggregation.

The spatial resolution of MODIS LST is about 1 km, whereas that of SMOS or SMAP SM is about tens of kilometers. It is hard to compare the remotely sensed LST and SM at the same spatial scale. Fortunately, there are some airborne L-band measurements such as that from the Passive/Active L-band Sensor (PALS) [28,29,30]. The PALS instrument is a simulator for SMAP, which includes both passive and active L-band sensors that view the land surface at a constant incidence angle of 40° [28,30]. It has been used in several major soil moisture experiments, including SMAP Validation Experiment 2008 (SMAPVEX08), 2012 (SMAPVEX12), 2015 (SMAPVEX12), and 2016 Manitoba (SMAPVEX16 Manitoba). Among them, only SMAPVEX12 was provided for public research, when we conducted this study, with gridded soil moisture data that was retrieved from the PALS instrument. The PALS SM data in SMAPVEX12 has a flight overpass sampling area of approximately 46 km × 47 km and a spatial resolution of ~1.5 km, which approximates to the spatial resolution of MODIS LST. Consequently, the primary objectives of this study are three-fold. First, this study aims to evaluate the spatial dynamics of PALS SM with MODIS LST from Terra/Aqua day/night as well as the difference between day and night. Secondly, the spatial dynamics of PALS SM with LST-derived SMIs are evaluated where the LST-derived SMIs are determined according to the theoretical boundaries of LST/FVC space using MODIS data and gridded meteorological data. Third, this study attempts to evaluate the performance of LST as a downscaling factor in the disaggregation of coarse-resolution SM.

## 2. Materials

Table 1 lists all of the materials used in this study. They are collected from three sources, i.e., NLDAS (or North American Land Data Assimilation System), MODIS, and SMAPVEX12. Specific information about these materials is provided in the following sections.

### 2.1. SMAPVEX12

**Figure 1:**
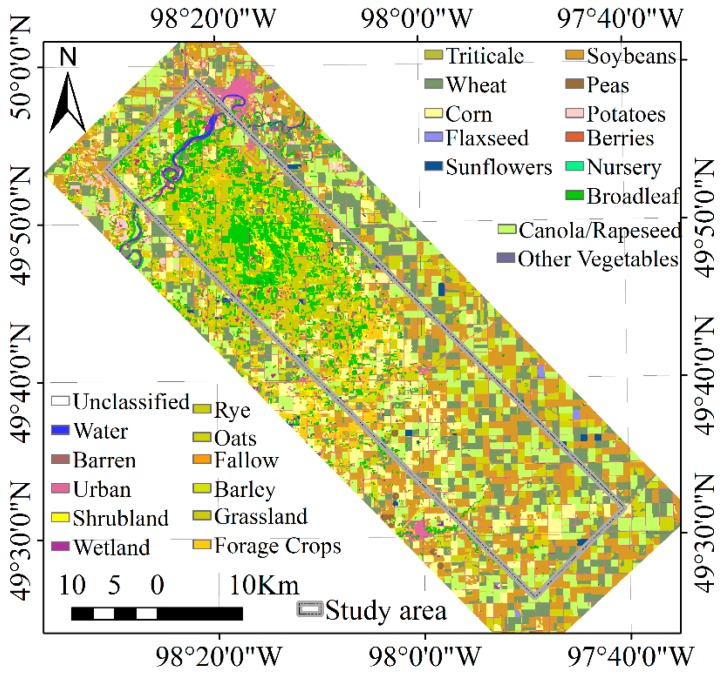
Spatial locations and land cover types of the study area in SMAPVEX12.

SMAPVEX12 is an aircraft-based field experiment conducted in Canada in order to develop and validate soil moisture algorithms of SMAP satellite mission. This campaign lasted six weeks from 7 June to 19 July 2012. During this period, SMAPVEX12 provided airborne microwave radiometer and radar observations at L-band just like what the SMAP satellite can provide. Concurrent with the airborne acquisitions, ground crews collected soil moisture data and other related parameters. Through a lot of effort, various data of the SMAPVEX12 can now be obtained for public access from the National Snow and Ice Data Center (NSIDC). In this study, Passive Active L-band Sensor (PALS) Soil Moisture Data [31] in Version 1, were collected. The PALS Soil Moisture Data provides soil moisture and microwave brightness temperature maps at a spatial resolution of 1.5 km with Universal Transverse Mercator (UTM) World Geodetic System 1984 (WGS84) coordinates, Zone 14 N. The data has been validated and a Pearson correlation of 0.87, RMSD (root mean square difference) 0.058 m^3^/m^3^, bias −0.015 m^3^/m^3^, and ubRMSD (unbiased RMSD) 0.056 m^3^/m^3^ were reported. More specific information about the SMAPVEX12 can be found in several literature [29,32]. Figure 1 shows the study area of the SMAPVEX12, which is located in an agricultural region south of Winnipeg, Manitoba province, Canada. The land cover type data has a resolution of 20 m and were derived from satellite imagery as part of the SMAPVEX12. Most of the land cover types in the area are croplands in the southeast of the study area, while there are some forest and grassland in the northwest of the study area.

### 2.2. MODIS Products

Four MODIS products are utilized in this study. They are:

➢ MODIS/Terra and Aqua Albedo Daily L3 Global 500 m SIN Grid V006 (MCD43A3);
➢ MODIS/Aqua and Terra Land Surface Temperature/Emissivity Daily L3 Global 1 km SIN Grid V006 (MOD11A1 and MYD11A1);
➢ MODIS/Terra and Aqua Leaf Area Index/FPAR 8-Day L4 Global 500 m SIN Grid V006 (MCD15A2H);
➢ MODIS/Aqua Aerosol 5-Min L2 Swath 3 km (MYD04_3K).

Their spatiotemporal resolutions are presented in the Table 1.

### 2.3. NLDAS-2 Forcing Dataset

**Figure 2:**
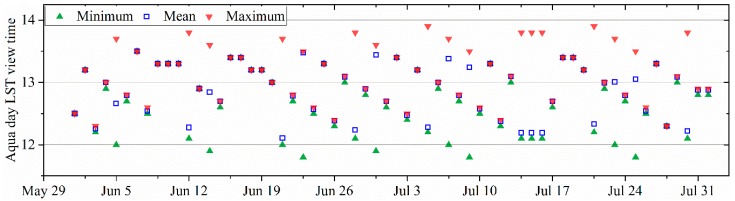
Local view time statistics of MODIS daytime LST on Aqua satellite over the SMAPVEX12 study area.

NLDAS-2 Forcing Dataset was selected because of its higher temporal (hourly) and spatial resolution (~12.5 km). This dataset is derived from the National Centers for Environmental Prediction (NCEP) North American Regional Reanalysis (NARR). NARR analysis fields are 32-km spatial resolution and 3-hourly temporal frequency. They are spatially interpolated to the NLDAS 1/8th-degree grid and then temporally disaggregated to the NLDAS-2 hourly frequency. Additionally, the meteorological variables such as surface pressure and air temperature are adjusted vertically to account for the vertical difference between the NARR and NLDAS fields of terrain height. More details about the processing methods are presented in [33]. The hourly land-surface forcing fields for NLDAS-2 are grouped into two GRIB files “File A” and “File B” where the former is utilized. Three variables in the file are required, including U/V-wind speed (m/s) at 10 m, air temperature (K) at 2 m, and downward shortwave radiation (W/m^2^). Note that the observing time recorded in NLDAS-2 file name is GMT time. It can be converted to local time by subtracting 6 according to the location of the study area. NLDAS-2 variables at 1 pm local time is selected to match with MODIS daytime LST on Aqua satellite according to the statistics in Figure 2.

## 3. Methods

**Figure 3:**
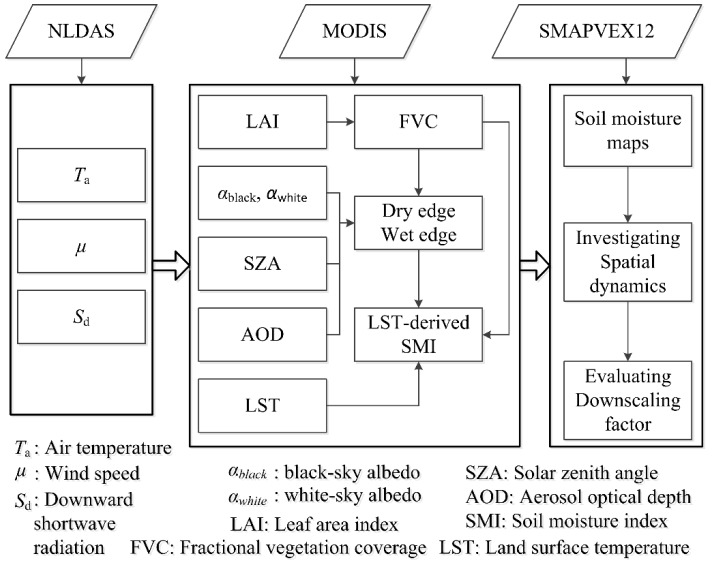
Flow of technique in this study.

Figure 3 presents the technical flow of this study. From NLDAS-2 Forcing dataset, we obtained necessary meteorological variables. From MODIS products, we obtained essential land surface parameters, including the LST and FVC. The meteorological variables and land surface parameters were combined to calculate dry and wet edges of LST/FVC space. Subsequently, LST-derived SMIs were determined according to the LST/FVC space. From SMAPVEX12, we obtained SM maps and then investigated its spatial dynamics with LST and LST-derived SMIs. Additionally, we evaluated LST as a downscaling factor. Three crucial parts within the technique flow are introduced as follows.

### 3.1. Determining Land Surface Parameters from MODIS Products

MOD11A1 and MYD11A1 were employed to obtain LST. Note that the obtained LST was filtered using the quality control file with criteria ‘pixel produced’, ‘good data quality’, ‘average emissivity error ≤0.02’, and ‘average LST error ≤ 2 K’ prior to application. MCD15A2H was used to obtain LAI (Leaf Area Index) and the following equation was used to calculate FVC:

(1) $$f_{v}=1-\exp\left(-k_{\mathrm{par}}\mathrm{LAI}\right)$$

where $f_{v}$ represents the FVC and $k_{\mathrm{par}}$ is the extinction coefficient assumed to take a value of 0.5 appropriate for canopies with spherical leaf angle distribution.

MCD43A3 was used to obtain land surface black-sky and white-sky albedos. Those albedos were utilized to calculate land surface blue-sky albedo and its disaggregation for soil and vegetation components with the help of MYD04_3K. Specifically, blue-sky albedo can be estimated as a linear combination of white and black-sky albedos:

(2) $$\alpha =f_{\mathrm{diff}}\alpha_{\mathrm{white}}+\left(1-f_{\mathrm{diff}}\right)\alpha_{\mathrm{black}}$$

where α is blue-sky albedo; $f_{\mathrm{diff}}$ is the fraction of diffuse skylight; $\alpha_{\mathrm{white}}$ and $\alpha_{\mathrm{black}}$ are white and black-sky shortwave albedo, respectively. $f_{\mathrm{diff}}$ can be determined by parsing a look-up table (LUT) of atmospheric radiative transfer model results. In this study, the LUT established with the help of 6S model was utilized. The LUT is indexed by solar zenith angle (0 to 89 degrees with a 1 degree step), optical depths (0 to 1.0 with 0.02 step), and aerosol model types (continental and maritime). Solar zenith angle and aerosol optical depth were obtained from the MYD04_3K product.

The shortwave radiation reaching a sensor can be assumed as the weighted sum of radiation coming from vegetation and soil substrate. This assumption has been validated in previous researches for separating surface albedo into vegetation and soil components [34,35,36]. Based on this assumption, the α over a vegetation/soil mixed surface can be considered as a linear combination of vegetation canopy albedo ($\alpha_{c}$) and bare soil albedo ($\alpha_{s}$) [36]:

(3) $$\alpha =f_{v}\alpha_{c}+\left(1-f_{v}\right)\alpha_{s}$$

In this study, α data over the pixels with FVC higher than 0.9 were averaged as $\alpha_{c}$. Subsequently, $\alpha_{s}$ is calculated by solving Equation (3).

### 3.2. Calculating LST-Derived Soil Moisture Index (SMI)

**Figure 4:**
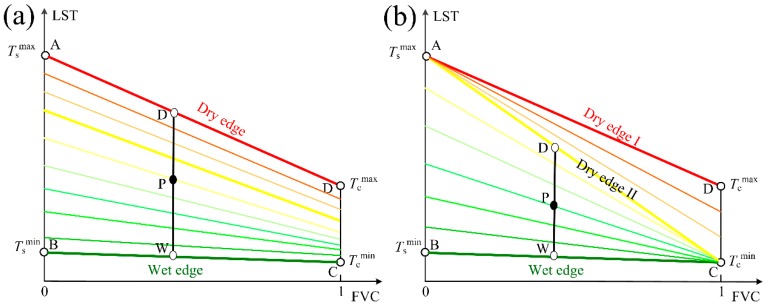
Two interpretations of the LST/FVC space: (**a**) conventional trapezoid and (**b**) two-stage trapezoid. Points A and D represent the soil and vegetation components under the maximum water stress with values of $T_{s}^{\max}$ and $T_{c}^{\max}$. Points B and C represent the soil and vegetation components under saturated water supply with values of $T_{s}^{\min}$ and $T_{c}^{\min}$. P is any given point. Points W and D represent LSTs on the wet and dry edges having the same FVC with point P.

The LST-derived SMIs, such as TVDI [37], VTCI [20], and SEE [11], etc. are defined by LST/FVC space [38,39]. The LST/FVC space is a conceptual model to demonstrate the correlation between SM and LST over heterogeneous land surface (mixed by dry/wet vegetation and soil) and under consistent atmospheric conditions [38]. Generally, there are three interpretations of the LST/FVC space i.e., the triangle model [39], the conventional trapezoid model [34], and the two-stage trapezoid model [18]. For the triangle model, vegetation is assumed to be under no water stress while soil can vary from wet to dry. In contrast, both the vegetation and soil can vary from wet to dry status for the conventional trapezoid and two-stage trapezoid models. However, the two-stage trapezoid is different from the conventional trapezoid in depicting the variations of soil and vegetation canopy temperatures (*T*_s_ and *T*_c_) with the variation of SM. Specifically, the two-stage trapezoid considers the response speed difference between *T*_s_ and *T*_c_ in view that vegetation can absorb deep soil moisture to maintain transpiration, whereas the conventional trapezoid does not [18]. Figure 4 illustrates the schematic diagram of the conventional trapezoid and the two-stage trapezoid. Lines within the LST/FVC space are isopiestic lines of soil moisture availability [38,39]. Points A, B, C, and D are called as temperature endmembers of the LST/FVC space. Line BC and AD are the so-called wet edge and dry edge, respectively. Please note that the lower triangle ABC in the two-stage trapezoid is the triangle model and Line AC is the dry edge of triangle model (i.e., dry edge II).

Based on the physical meaning of the LST/FVC space, the relative distance of a given point’s LST (see point P in Figure 4) to the corresponding LSTs for wet (see point W) and dry conditions (see point D) under the same FVC can indicate the extent of SM. Therefore, the SMI generally have the following expression:

(4) $$\mathrm{SMI}=\frac{T_{s}^{\max}-T_{s}}{T_{s}^{\max}-T_{s}^{\min}}$$

where LST is the remotely sensed land surface temperature (K) for a given pixel; LST_dry_ and LST_wet_ are the LST on the dry and wet edges with the same FVC of that pixel; *T*_s_ is the soil temperature of that pixel; *T*_s_^max^ and *T*_s_^min^ are the soil temperatures corresponding to the dry edge and wet edge conditions. SMI varies from 0 to 1 where 0 represents very dry condition and 1 represents very wet condition. Note that it is also very common to see that 1−SMI is used as soil moisture indicator.

To calculate the SMI, soil component temperature (i.e., *T*_s_) and the temperature endmembers (i.e., *T*_s_^max^, *T*_s_^min^, *T*_c_^max^, and *T*_c_^min^) are required. For obtaining *T*_s_, the conventional trapezoid is different from the two-stage trapezoid. Taking the point P in Figure 4 for instance, the following expressions are used for obtaining *T*_s_ based on the conventional trapezoid:

(5) $$T_{s}=T_{s}^{\max}-\frac{{\mathrm{LST}}_{D}-\mathrm{LST}}{{\mathrm{LST}}_{D}-{\mathrm{LST}}_{W}}\left(T_{s}^{\max}-T_{s}^{\min}\right)$$

(6) $${\mathrm{LST}}_{D}=\left(T_{c}^{\max}-T_{s}^{\max}\right)\times f_{v}+T_{s}^{\max}$$

(7) $${\mathrm{LST}}_{W}=\left(T_{c}^{\min}-T_{s}^{\min}\right)\times f_{v}+T_{s}^{\min}$$

where LST and $f_{v}$ are remotely sensed LST and FVC for the point P; ${\mathrm{LST}}_{D}$ and ${\mathrm{LST}}_{W}$ are the temperatures of point D and W. With regard to the two-stage trapezoid, Equation (6) is changed into Equation (8) when the point P’s LST is less than the ${\mathrm{LST}}_{D}$:

(8) $${\mathrm{LST}}_{D}=\left(T_{c}^{\min}-T_{s}^{\max}\right)\times f_{v}+T_{s}^{\max}$$

If the point P’s LST ≥ ${\mathrm{LST}}_{D}$ within the two-stage trapezoid, $T_{s}$ is equal to $T_{s}^{\max}$.

There are two theoretical methods proposed by Sun [40,41] and Long [34] to determine the temperature endmembers of LST/FVC space. Sun’s method combined the Priestley-Taylor equation and the land surface energy balance. Specifically, the Priestley-Taylor equation was used to determine latent heat flux (LE, W/m^2^) on the dry and wet edges by setting the Priestley-Taylor parameter as zero and an empirical maximum of 1.26. Finally, the four temperature endmembers can be determined as:

(9) $$\left\{ \begin{array}{l} T_{c}{}^{\max}=\frac{\left(1-\alpha_{c}\right)S_{d}+\varepsilon_{c}\varepsilon_{a}\sigma T_{a}{}^{4}-\varepsilon_{c}\sigma T_{a}{}^{4}}{4\varepsilon_{c}\sigma T_{a}{}^{3}+\rho c_{p}/r_{a}{}^{c}}+T_{a}\\ T_{s}{}^{\max}=\frac{\left(1-\alpha_{s}\right)S_{d}+\varepsilon_{s}\varepsilon_{a}\sigma T_{a}{}^{4}-\varepsilon_{s}\sigma T_{a}{}^{4}}{4\varepsilon_{s}\sigma T_{a}{}^{3}+\rho c_{p}/\left[r_{a}{}^{s}\left(1-n_{s}\right)\right]}+T_{a}\\ T_{c}{}^{\min}=\frac{\left(1-\alpha_{c}\right)S_{d}+\varepsilon_{c}\varepsilon_{a}\sigma T_{a}{}^{4}-\varepsilon_{c}\sigma T_{a}{}^{4}}{4\varepsilon_{c}\sigma T_{a}{}^{3}+\rho c_{p}/\left[r_{a}{}^{c}\left(1-\phi^{\max}\frac{\Delta }{\Delta +\gamma }\right)\right]}+T_{a}\\ T_{s}{}^{\min}=\frac{\left(1-\alpha_{s}\right)S_{d}+\varepsilon_{s}\varepsilon_{a}\sigma T_{a}{}^{4}-\varepsilon_{s}\sigma T_{a}{}^{4}}{4\varepsilon_{s}\sigma T_{a}{}^{3}+\rho c_{p}/\left[r_{a}{}^{s}\left(1-n_{s}\right)\left(1-\phi^{\max}\frac{\Delta }{\Delta +\gamma }\right)\right]}+T_{a} \end{array}\right.$$

where $T_{a}$ is near surface air temperature (K); $\alpha_{c}$ and $\alpha_{s}$ are shortwave (0.3–5.0 μm) albedos of vegetation component and soil component (unitless); $\varepsilon_{c}$ and $\varepsilon_{s}$ are broadband emissivities (8~14 μm) of vegetation component and soil component (unitless). $\varepsilon_{c}$ and $\varepsilon_{s}$ can be obtained from look-up tables of [42] in the case of no measurements; For simplification, the average $\varepsilon_{c}$ and $\varepsilon_{s}$ from the look-up tables [42] were employed, which are 0.983 and 0.959, respectively. $r_{a}{}^{c}$ and $r_{a}^{s}$ are aerodynamic resistances above vegetation canopy and soil component surface (s/m). Please see Appendix A for the determination of $r_{a}{}^{c}$ and $r_{a}^{s}$; $n_{s}$ is a fraction coefficient (unitless) with a default value of 0.35 [34,41]. $\varepsilon_{a}$ is atmospheric emissivity (unitless), which can be approximately determined by air temperature [43] i.e., $\varepsilon_{a}=1.0-0.261\exp\left[-7.77\times 10^{-4}{\left(273-T_{a}\right)}^{2}\right]$. $S_{d}$ is down-welling shortwave radiation (W/m^2^). σ is the Stefan–Boltzmann constant with a value about 5.67 × 10^−8^ W/(m^2^∙K^4^). ρ is air density (kg/m^3^), which can be approximately set as 1.225 kg/m^3^. $c_{p}$ is specific heat of air, which can be approximately set as 1006 J/(kg∙K). ∆ represents the slope of the saturated vapour pressure-temperature relation. γ is psychrometric constant. The term Δ/(Δ+γ) mainly depends on air temperature, therefore, it can be calculated as [44]: $\Delta /\left(\Delta +\gamma \right)=0.0127\times \left(T_{a}-273.15\right)+0.3464$. $\phi^{\max}$ is the Priestley-Taylor parameter for very wet condition and an overall mean value of 1.26 is selected [45,46]. Long’s method is the same as the above Sun’s method in calculating $T_{c}^{\max}$ and $T_{s}^{\max}$, whereas they are different in determining $T_{c}^{\min}$ and $T_{s}^{\min}$. The following equation is used in the Long’s method:

(10) $$T_{s}^{\min}=T_{c}^{\min}=T_{a}$$

### 3.3. Evaluating LST As a Downscaling Factor

As mentioned, there are generally three categories of optical/thermal and microwave fusion methods—statistical regression, relative ratio, and physical model for simplification [4]. Although they have different expressions, they have similarity in the sense that the relationship between SM and downscaling factors is constructed at coarse resolution and then that relationship is applied at a finer resolution [4]. We selected the following typical statistical regression to evaluate LST as downscaling factors because, (1) it is easy to be realized as compared with the relative ratio and physical model; (2) it originates from the LST/FVC space [39], which has a similar basis with the relative ratio and physical model; and (3) such a method has great potential for operational disaggregation of coarse-resolution SM [47].

(11) $$SM=\sum_{i=0}^{2}\sum_{j=0}^{2}a_{ij}{\mathrm{FVC}}^{*}{}^{i}{\mathrm{LST}}^{*}{}^{j}$$

where ${\mathrm{FVC}}^{*}$ and ${\mathrm{LST}}^{*}$ are normalized downscaling factors. $a_{ijk}$ are the coefficients. Please note that the downscaling factors should be normalized into the 0–1 range with min-max normalization. Initially, only two downscaling factors (normally LST and NDVI) were used. Subsequently, the third downscaling factor albedo or microwave brightness was added. Here, we utilize two factors i.e., LST and FVC according to our aims to evaluate LST as a downscaling factor. The degree of agreement between simulated and original SM was measured by Pearson’s correlation coefficient and root-mean-square-error (RMSE). For the Pearson correlations, the 2-tailed test of significance was used. Since the relationship is constructed at coarse resolution and then applied at a finer resolution for downscaling, the agreement between simulated and original SM can indicate the performance of downscaling factors. Higher agreement demonstrates higher performance and vice versa.

## 4. Results

### 4.1. PALS Soil Moisture

**Figure 5:**
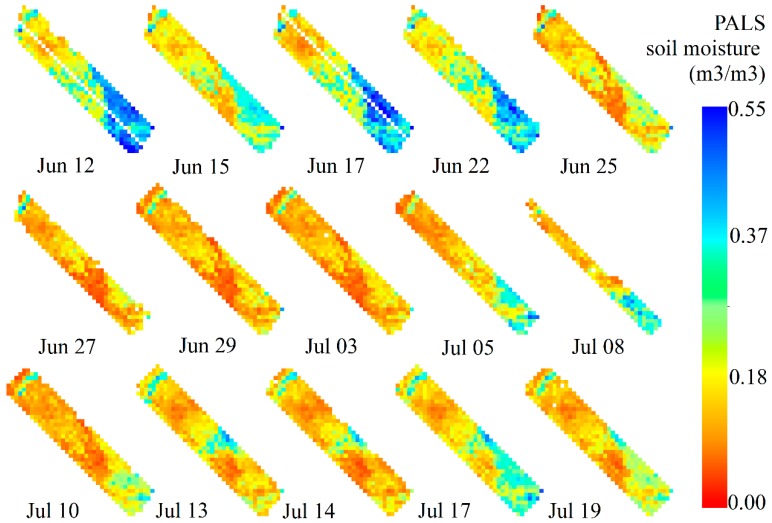
Maps of PALS soil moisture (m^3^/m^3^) during the SMAPVEX12 experiment.

Figure 5 illustrates all of the PALS SM data. Maps of PALS SM are rendered in uniform data range from 0 to 0.55 m^3^/m^3^. From a perspective of spatial distribution, the study area is relatively wet in the southeast part, while it is relatively dry in the northwest part. From a perspective of time, the study area is wet at the beginning of the experiment and experiences a drying process during the next experiment.

**Figure 6:**
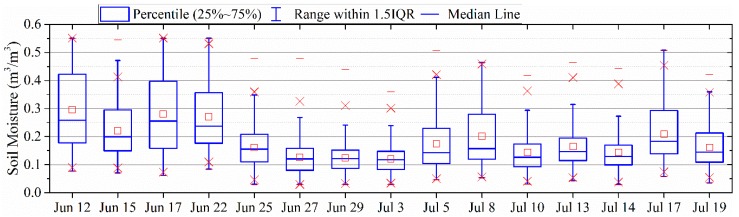
Box chart of the PALS soil moisture (m^3^/m^3^) during the SMAPVEX12 experiment.

A box chart of the PALS SM data is shown in Figure 6, which quantitatively illustrates the temporal variation of SM. In the box plots, the central rectangle spans the first quartile (Q1) to the third quartile (Q3). A segment inside the rectangle shows the median, and “whiskers” above and below the box show the minimum and the maximum values within Q1 − 1.5 × IQR and Q3 + 1.5 × IQR, where IQR represents Inter Quartile Range (i.e., Q3−Q1). The little square within the rectangle represents the mean of the data. The median SM of this study area reached up to 0.25 m^3^/m^3^ on June 12, 17, and 22. The median SM then decreases over the next few days and its minimum closes to 0.1 m^3^/m^3^ on 3 July. The temporal variation is definitely not steady. There are days when SM temporarily increases during the drying process from a wet beginning to the end of the experiment.

### 4.2. Spatial Dynamics of SM with LST

**Figure 7:**
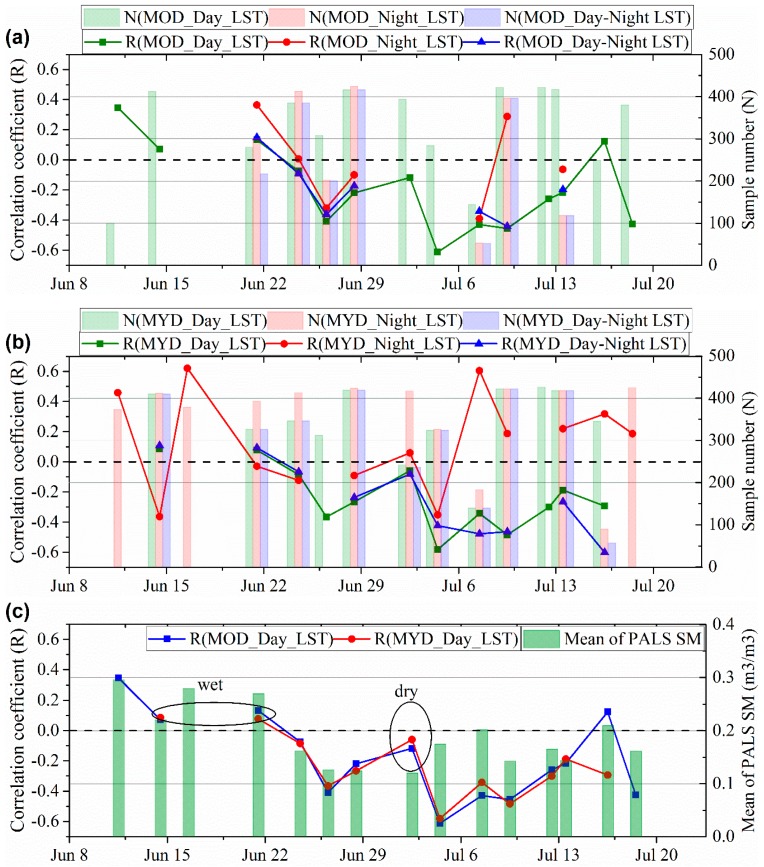
Spatial correlations of PALS soil moisture with day, night, and day-night differential LST from (**a**) Terra MODIS and (**b**) Aqua MODIS. Daytime LSTs from Terra and Aqua MODIS are compared specially in subgraph (**c**). MOD and MYD represent the MODIS sensors on Terra and Aqua satellites, respectively. Here, R(*) represent Pearson correlation coefficient between * and SM. N(*) represents the number of comparison samples between * and SM.

Spatial correlations between LST and PALS SM are shown in Figure 7, where subgraph (a) is for MODIS day, night, and day-night differential LST on Terra satellite; subgraph (b) is those on Aqua satellite; and subgraph (c) compares the Terra daytime LST with the Aqua daytime LST in particular. The N in Figure 7 is the number of comparison samples. The higher degree of relevance indicates closer correlation between LST and SM. The mean of PALS SM is the average value of SM over the study area with the unit of m^3^/m^3^.

Figure 7 indicates that MODIS daytime LST, on either the Terra or the Aqua satellite, presents anti-correlation with SM at most times. In contrast, the nighttime LST presents positive correlation some days and anti-correlation on other days. Moreover, the degree of relevance is bigger for daytime LST at most times as compared with the nighttime LST. The day-night differential LST does not present closer correlation with SM when compared with the daytime LST. Besides, it is harder to obtain the day-night differential LST because of cloud contamination. Therefore, daytime LST is better than nighttime LST and the day-night differential LST to depict the spatial dynamics of SM. In view of this, we compared daytime LST from Terra and that from Aqua with the PALS SM (see Figure 7c). Comparison results indicate very similar performance between Terra and Aqua daytime LST. There are no absolute advantages to conclude that Aqua daytime LST outperforms the Terra daytime LST in depicting the spatial variation of SM.

**Figure 8:**
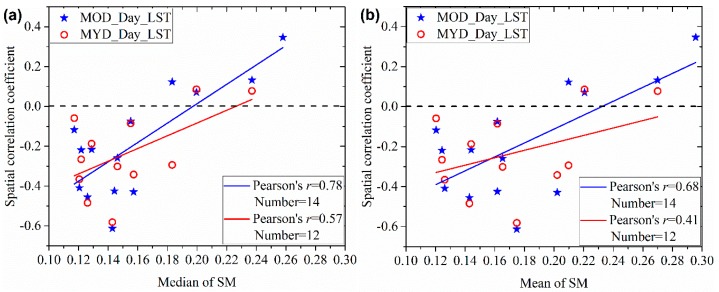
Comparing (**a**) Median and (**b**) Mean of SM over the study area with the spatial correlation coefficient between daytime LST and SM. MOD_Day_LST and MYD_Day_LST are the daytime LSTs from Terra and Aqua MODIS sensors, respectively. Pearson’s *r* is the Pearson correlation coefficient and Number is the number of samples.

In addition, we found high variability in the degree of relevance in Figure 7. For most cases, the daytime LST presents negative and significant (with a *p*-value = 0.05) correlation with SM. However, the daytime LST presents positive or insignificant correlation with SM at the beginning of the experiment. From Figure 5 and the mean SM of the study area, we know that the study area is wet in the experiment’s initialization phase and it experiences a drying process during the next period of the experiment. Therefore, the magnitude of SM may have a significant influence on the spatial correlation between SM and daytime LST. Figure 8 shows the scatter plots between spatial correlation coefficients of daytime LST with SM and median or mean value of the PALS SM. This figure demonstrates the influence of SM on its spatial correlation with daytime LST. When SM is relatively low, the spatial correlation tends to be significant (negative), indicating SM-LST coupling. When SM is high, the spatial correlation tends to be insignificant, indicating SM-LST decoupling. In addition, we found that the SM-LST link was very weak on 3 July, also indicating SM-LST decoupling, when the study area is very dry (see Figure 7). Consequently, our results indicated that very wet and very dry conditions could both cause SM-LST decoupling.

### 4.3. Spatial Dynamics of SM with LST-Derived SMI

**Figure 9:**
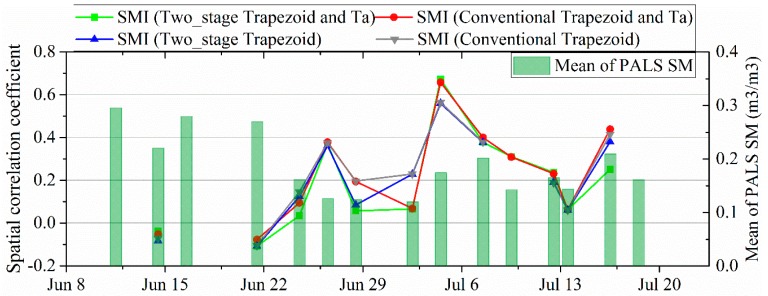
Spatial correlation between LST-derived SMIs and PALS SM. The SMI (two-stage Trapezoid) and (Conventional Trapezoid) are two SMIs based on the two-stage trapezoid and conventional trapezoid with endmembers determining by Sun’s method. The SMI (two-stage Trapezoid and Ta) and (Conventional Trapezoid and Ta) are the other two SMIs based on the two-stage trapezoid and conventional trapezoid with endmembers determining by Long’s method.

Figure 9 shows the spatial correlation between LST-derived SMIs and PALS SM, where daytime LST from Aqua MODIS was used. Four SMIs were calculated based on different LST/FVC space models. The SMI (Two-stage Trapezoid) and (Conventional Trapezoid) are two SMIs based on the two-stage trapezoid and conventional trapezoid with endmembers determining by Sun’s method [40,41]. The SMI (Two-stage Trapezoid and Ta) and (Conventional Trapezoid and Ta) are the other two SMIs based on the two-stage trapezoid and conventional trapezoid with endmembers determining by Long’s method [34]. Although different LST/FVC spaces were used, the LST-derived SMIs presented similar spatial correlations with the PALS SM during the SMAPVEX12 experiment. In general, the SMIs have a positive correlation with SM. However, the degree of relevance is unsteady during the whole period of experiment. We can see that the spatial correlation is insignificant at the beginning of the experiment when the study area is relatively wet. With the drying of the study area, the spatial correlation becomes positive and significant (with a *p*-value = 0.05). However, the spatial correlation becomes weak and even insignificant on some days when the study area is very dry such as on 3 July and 14 July.

**Figure 10:**
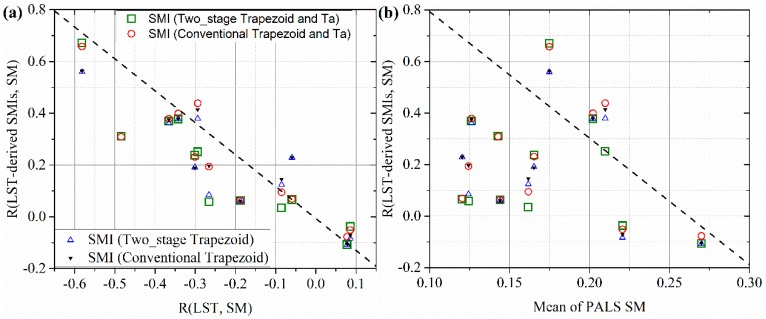
Evaluating the influence of SM and LST-SM link on the LST-derived SMI-SM link. (**a**) R(LST, SM) is the spatial correlation coefficient between LST and (**b**) SM. R(LST-derived SMIs, SM) is the spatial correlation coefficient between LST-derived SMIs and SM.

We also compared the spatial correlation of LST-derived SMI-SM link with that of LST-SM link and the mean of PALS SM using scatter plots. The comparison results are shown in Figure 10, which shows significant relationship between LST-SM link and LST-derived SMI-SM link. Significant positive correlation between SMI and SM appeared when significant negative correlation between LST and SM occurs and vice versa. Likewise, Figure 10 indicated that LST-derived SMI-SM link is also influenced by the magnitude of SM. Significant positive correlation between LST-derived SMI and SM appeared when SM is relatively low but not very low or in other words in transitional condition [24]. When the study area is very dry or very wet, weak LST-derived SMI and SM link occurs. Therefore, the SM magnitude of the study area influences not only the LST-SM link but also the LST-derived SMI-SM link. Very wet or very dry status of the study area would induce a decoupling effect between SM and LST as well as LST-derived SMI.

### 4.4. Evaluating LST As a Downscaling Factor

**Figure 11:**
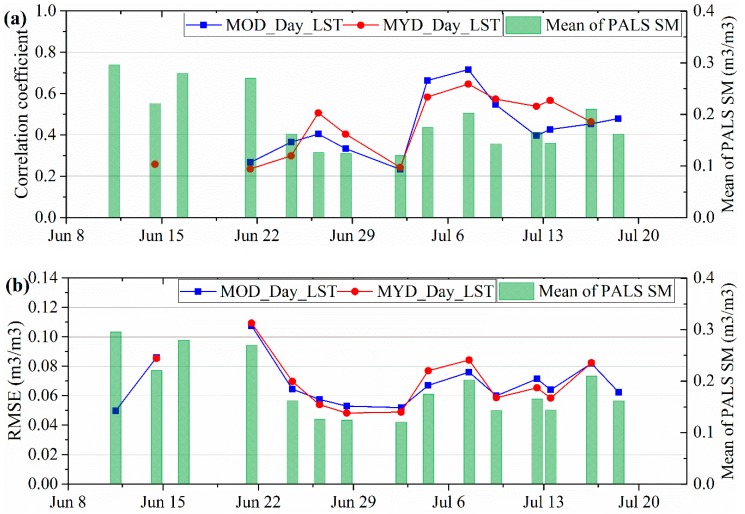
Results of evaluating LST as a downscaling factor. (**a**) and (**b**) are the Pearson correlation coefficient and RMSE (m^3^/m^3^) between PALS SM and the simulated SM based on LST.

Figure 11 shows the results of evaluating LST as a downscaling factor, where MOD_Day_LST and MYD_Day_LST represented daytime LSTs on board the Terra and Aqua satellites, respectively. The correlation coefficient belongs to the Pearson correlation and 2-tailed test of significance was used. Results indicated that all of the correlations are significant at the 0.05 level. MOD_Day_LST and MYD_Day_LST have very similar performances as downscaling factors in simulating SM. Additionally, we found the unsteady variation in the performances of the daytime LST as a downscaling factor.

**Figure 12:**
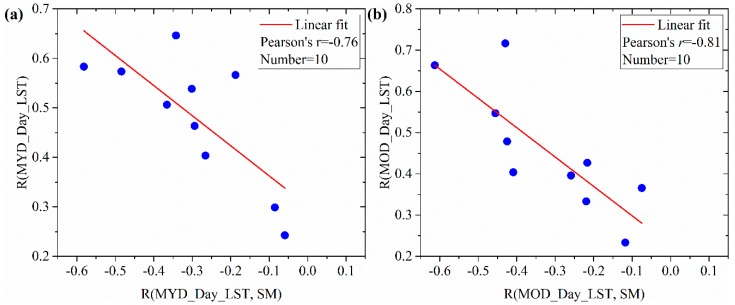
Evaluating the influence of LST-SM decoupling effect on the performance of LST as a downscaling factor. (**a**) R(MYD_Day_LST, SM) and R(MOD_Day_LST, SM) are the Pearson correlations between daytime LSTs and SM. R(MYD_Day_LST) and (**b**) R(MOD_Day_LST) are Pearson correlations between original SM and simulated SM based on the downscaling factors of MYD_Day_LST and MOD_Day_LST.

In order to interpret the unsteady variation, we compared the LST-SM link with the LST performance in simulating SM. Results are shown in Figure 12 where the horizontal axis is the correlation coefficient of daytime LST with SM i.e., R(MYD_Day_LST, SM) and R(MOD_Day_LST, SM). The vertical axis is the correlation coefficient of original SM with simulated SM based on the downscaling factors of MYD_Day_LST and MOD_Day_LST i.e., R(MYD_Day_LST) and R(MOD_Day_LST). Positive R(MYD_Day_LST, SM) or R(MOD_Day_LST, SM) is excluded in the comparison since it implies SM-LST decoupling. The results demonstrate that the performance of LST as a downscaling factor is significantly correlated with the LST-SM link. When LST-SM link is closely related, the LST presents higher performance in simulating SM and vice versa. In other words, the decoupling effect of LST-SM link can reduce the performance of LST as a downscaling factor.

## 5. Discussion

### 5.1. Differences between Temporal and Spatial Evaluation

Gaining an understanding of the LST-SM link and LST-derived SMI-SM link from observations could help refine the disaggregation of coarse-resolution SM. Recently, Pablos et al. (2016) conducted temporal evaluation of SM and LST dynamics [10,27]. Several interesting phenomena have been found, such as that a strong anti-correlation is observed between SM and maximum LST at the daily scale. This conclusion has been used in downscaling microwave soil moisture with optical/thermal remote sensing data. For example, Portal et al. (2018) selected daytime Aqua MODIS LST since it is closer (in time) to the daily maximum LST than other available MODIS LST products [47]. On a seasonal scale, results show a stronger anti-correlation in autumn, spring and summer indicates SM-LST coupling, than in winter indicating SM-LST decoupling [10]. These conclusions were made from the perspective of temporal evaluation. Specifically, all 1-km MODIS LST pixels within the corresponding SMOS pixels were averaged to investigate a temporal SM-LST relationship [10].

By contrast, we evaluated the SM-LST relationship from the perspective of spatial comparison. Moreover, LST-derived SMI based on LST/FVC space is a widely used downscaling factor. We calculated and evaluated it by combining gridded meteorological data and remote sensing data. We found better agreement between SM and daytime LST than the nighttime and the day-night differential LST. As regarding the daytime LST on Aqua and Terra, there are no absolute advantages to demonstrate that SM exhibits stronger anti-correlation to MODIS daytime LST on Aqua than that on Terra, although Aqua is closer to the daily maximum LST in time. In addition, Terra and Aqua daytime LST have very similar performances in simulating SM as downscaling factors. We think this is a difference between spatial evaluation and temporal evaluation. From the temporal evaluation, Aqua daytime LST should present greater agreement with SM. However, from the spatial evaluation, Aqua and Terra daytime LSTs present very similar agreement with SM.

Besides, we found that the magnitude of SM can strongly correlate with the decoupling effect on the SM-LST link and the SM-LST derived SMI link in addition to the seasonal variation causing the SM-LST decoupling [10]. When the SM of the study area is in transitional condition, the spatial correlation between SM and LST tends to be more significant (negative), indicating SM-LST coupling. When the study area is in very wet or very dry, the spatial correlation tends to be insignificant, indicating SM-LST decoupling. Likewise, the spatial correlation between SM and LST-derived SMI is insignificant when the study area is very wet or very dry, indicating SM-LST derived SMI decoupling. Under the transitional condition, the spatial correlation between SM and LST-derived SMI becomes more significant, indicating SM-LST derived SMI coupling.

### 5.2. Understanding the Coupling and Decoupling Effect

From the perspective of spatial variation, three types of factors influence LST or soil surface temperature [18]: (1) Factors that influence solar irradiation energy impacting the surface, including latitude, altitude, and season, etc.; (2) factors that influence the thermal characteristics of land surface, such as soil moisture and vegetation coverage; and (3) factors that influence the interaction of water and heat between the surface and the atmosphere, including wind speed, air temperature, and air humidity, etc. For our relatively small study area, the above first and third types of factors can be regarded as homogeneous. The factors that influence the thermal characteristics of land and soil surface are the dominated factors, which include SM and vegetation coverage. The latter can be circumvented using the LST-derived SMI calculated from the LST/FVC space. Consequently, SM is the main factor dominating the above decoupling phenomenon on SM-LST link as well as on SM-LST derived SMI link in our study area.

The above decoupling phenomenon can be further interpreted by the SM and ET regimes [24]. First, the higher rate of ET indicates more heat consumption on surfaces. Therefore, the spatial dynamic of ET rate can determine the spatial dynamic of surface temperature within a relative small area. Based on this acknowledge, it is easy to understand that SM is strongly linked with LST and LST-derived SMI when SM is the limited factor for ET (i.e., SM limited regime). Correspondingly, decoupling effect appears when energy is the limited factor (i.e., energy limited regime). According to the conceptual framework for defining ET regimes as a function of SM, evaporative fraction is dependent of the SM when SM is greater than the wilting point ($\theta_{\mathrm{WILT}}$) and less than a given critical SM value ($\theta_{\mathrm{CRIT}}$). When SM lies above the $\theta_{\mathrm{CRIT}}$ (very wet), the study area is in an energy limited ET regime. In addition, it is notable that no ET takes place anymore when SM is below $\theta_{\mathrm{WILT}}$ (very dry). For this condition, a decoupling effect of SM to LST and LST-derived SMI also occurs. These are the main reasons causing the decoupling effect under very dry and very wet conditions.

### 5.3. Improving the Decoupling Effect

Our results have indicated that a decoupling effect can reduce the performance of LST for downscaling SM. Since the decoupling effect can be interpreted by the SM and ET regimes, we think the surface net incoming radiation (R_n_) is a significant complementary downscaling factor representing energy portion in an energy limited ET regime. Therefore, R_n_ was added to LST and FVC for simulating SM:

(12) $$SM=\sum_{i=0}^{2}\sum_{j=0}^{2}\sum_{k=0}^{2}a_{ijk}{\mathrm{FVC}}^{*}{}^{i}{\mathrm{LST}}^{*}{}^{j}R_{n}{}^{*k}$$

where $R_{n}{}^{*}$ is min-max normalized R_n_. Here, the R_n_ was estimated using the following method [43]:

(13) $$\begin{array}{l} R_{n}=\left(1-\alpha \right)\times S_{d}+\left(\varepsilon_{a}-\varepsilon \right)\times \sigma \times T_{a}{}^{4}\\ \varepsilon =0.97 \end{array}$$

where ε is broadband land surface emissivity. The other variables have the same meanings as that in Equations (3) and (9).

**Figure 13:**
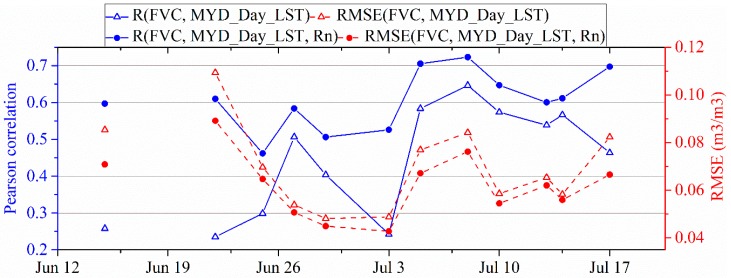
Pearson correlation coefficient (R) and RMSE between PALS SM and simulated SM that is generated by FVC and MOD_Day_LST (FVC, MOD_Day_LST) or FVC, MYD_Day_LST, and R_n_ (FVC, MYD_Day_LST, R_n_).

Figure 13 compares the performances in simulating SM using FVC and daytime LST as downscaling factor with that using FVC, daytime LST, and R_n_ as downscaling factors. Pearson correlation coefficients of the simulation with R_n_ i.e., R(FVC, MYD_Day_LST, R_n_) were greater than that of the simulation without R_n_ i.e., R(FVC, MYD_Day_LST) during the whole experiment period. Likewise, RMSE(FVC, MYD_Day_LST, R_n_) was lower than RMSE(FVC, MYD_Day_LST) during the whole experiment period. Adding R_n_ as a downscaling factor to FVC and daytime LST improves performance in simulating SM. However, adding R_n_ does not cover the decoupling effect causing by the very dry condition in theory. For the very dry condition, soil texture, structure, or type play a significant role in affecting soil surface temperature [18]. Future downscaling algorithms should consider these factors.

## 6. Conclusions

We conducted a spatial evaluation of SM, LST, and LST-derived SMIs link during SMAPVEX12. Aerial PALS SM data and MODIS day/night LSTs on Terra and Aqua were used. In addition, NLDAS-2 gridded meteorological data was employed to calculate the dry and wet edges as well as the LST-derived SMIs.

Through the evaluation, we found better agreement between SM and daytime LST than the nighttime and the day-night differential LST. As regarding the daytime LST on Aqua and Terra, they present very similar spatial agreement with SM. In addition, Terra and Aqua daytime LST have very similar performances in simulating SM as downscaling factors. Besides, we found that the magnitude of SM strongly correlate with the decoupling effect of the SM-LST link and the SM-LST derived SMI link. When the SM of the study area is in transitional condition, SM is coupled with the LST and LST-derived SMI. By contrast, when the study area is in very wet or very dry condition, decoupling effect occurs. Further evaluation demonstrated that the decoupling effect reduces the performance of LST as a downscaling factor. The decoupling effect can be interpreted by the conceptual definition of ET regimes as a function of SM. For very wet condition, the study area is in an energy limited ET regime rather than a SM limited ET regime. For very dry condition, no ET takes place. Therefore, decoupling effect occurs when the study area is in very wet or very dry condition.

The decoupling effect challenges the algorithms based on LST or LST-derived SMIs for downscaling coarse-resolution SM. We have found that adding R_n_ to LST and FVC can improve the adverse influence of decoupling effect for very wet conditions. The future downscaling algorithms should consider other factors such as soil texture, structure, or type in order to tackle the decoupling effect for very dry conditions.

## Figures and Tables

**Table 1:** Material information used in this study.

Sources	Parameters	Products	Spatial Resolution	Temporal Resolution
SMAPVEX12	Microwave soil moisture	PALS	1.5 km	/
MODIS	Black/white-sky albedos	MCD43A3	500 m	daily
	Solar zenith angle	MYD04_3K	3 km	5 min
	Aerosol optical depth			
	Leaf area index	MCD15A2H	500 m	8-day
	Land surface temperature	MOD11A1 MYD11A1	1 km	daily
NLDAS	Air temperature	NLDAS-2 Forcing FILE A	1/8th-degree	hourly
	Wind speed			
	Down shortwave radiation			

## Appendix A. Determining Aerodynamic Resistances for Vegetation and Soil

According to the Monin-Obukhov Similarity (MOS) theory, the aerodynamic resistance above vegetation canopy ($r_{a}{}^{c}$) and that above soil ($r_{a}^{s}$) can be expressed as:

(A1) $$r_{a}^{c}=\frac{1}{k^{2}\mu }\left[\ln\left(\frac{z_{m}-d}{z_{om}}\right)-\psi_{m\left(z_{m}\right)}+\psi_{m\left(z_{om}\right)}\right]\left[\ln\left(\frac{z_{T}-d}{z_{oh}}\right)-\psi_{h\left(z_{T}\right)}+\psi_{h\left(z_{oh}\right)}\right]$$

(A2) $$r_{a}^{s}=\frac{1}{0.0015\mu_{1m}},\;\mu_{1m}=\mu \frac{\ln\left(1/z_{om}\right)-\psi_{m\left(z_{m}\right)}+\psi_{m\left(z_{om}\right)}}{\ln\left(z_{m}/z_{om}\right)-\psi_{m\left(z_{m}\right)}+\psi_{m\left(z_{om}\right)}}$$

where k is the von Karman’s constant (0.41); μ is the wind speed (m/s) observed at the reference height of $z_{m}$ (m); $z_{T}$ is the reference height for air temperature observation (m); d is the zero plane displacement (m); $z_{om}$ is the roughness length for momentum transfer (m); $z_{oh}$ is the roughness length for heat transfer (m). Normally, there are assumptions that vegetation height $h_{c}$ = 1 m, therefore, $d=2h_{c}/3$, $z_{om}=h_{c}/10$, and $z_{oh}=z_{om}/7$ for vegetation. For soil, $z_{om}$ can be taken as 0.005 m. $\psi_{m\left(z_{m}\right)}$ and $\psi_{m\left(z_{om}\right)}$ are the stability correction factors for momentum transfer (unitless) at the heights of $z_{m}$ and $z_{om}$, respectively. $\psi_{h\left(z_{T}\right)}$ and $\psi_{h\left(z_{oh}\right)}$ are the stability correction factors for heat transfer (unitless) at the heights of $z_{T}$ and $z_{oh}$, respectively.

To determine these stability correction factors, the atmosphere stability is firstly measured by the Monin-Obukhov length:

(A3) $$L=-\frac{\rho c_{p}\mu_{*}^{3}T_{a}}{kgH}$$

where L is the Monin-Obukhov length (m); $\mu_{*}$ is the friction velocity (m/s); g is the acceleration of gravity (9.8 m/s); H is the sensible heat flux (W/m^2^). $\mu_{*}$ can be calculated from the given μ:

(A4) $$\mu_{*}=k\mu /\left[\ln\left(\frac{z_{m}-d}{z_{om}}\right)-\psi_{m\left(z_{m}\right)}+\psi_{m\left(z_{om}\right)}\right]$$

The expression of H in Formula (A3) depends on the conditions of land surface. Specifically, the H for dry/wet vegetation/soil ($H_{\mathrm{dry}}^{c}$, $H_{\mathrm{wet}}^{c}$, $H_{\mathrm{dry}}^{s}$, and $H_{\mathrm{wet}}^{s}$) can be calculated as:

(A5) $$\left\{ \begin{array}{l} H_{\mathrm{dry}}^{c}=\left(1-\alpha_{c}\right)S_{d}+\varepsilon_{c}\varepsilon_{a}\sigma T_{a}{}^{4}-\varepsilon_{c}\sigma {\left(T_{c}{}^{\max}\right)}^{4}\\ H_{\mathrm{wet}}^{c}=\left(1-\phi^{\max}\frac{\Delta }{\Delta +\gamma }\right)\left[\left(1-\alpha_{c}\right)S_{d}+\varepsilon_{c}\varepsilon_{a}\sigma T_{a}{}^{4}-\varepsilon_{c}\sigma {\left(T_{c}{}^{\min}\right)}^{4}\right]\\ H_{\mathrm{dry}}^{s}=\left(1-n_{s}\right)\left[\left(1-\alpha_{s}\right)S_{d}+\varepsilon_{s}\varepsilon_{a}\sigma T_{a}{}^{4}-\varepsilon_{s}\sigma {\left(T_{s}{}^{\max}\right)}^{4}\right]\\ H_{\mathrm{wet}}^{s}=\left(1-n_{s}\right)\left(1-\phi^{\max}\frac{\Delta }{\Delta +\gamma }\right)\left[\left(1-\alpha_{s}\right)S_{d}+\varepsilon_{s}\varepsilon_{a}\sigma T_{a}{}^{4}-\varepsilon_{s}\sigma {\left(T_{s}{}^{\min}\right)}^{4}\right] \end{array}\right.$$

For L < 0, it is under unstable conditions:

(A6) $$\psi_{m\left(z_{i}\right)}=2\ln\left(\frac{1+x_{\left(z_{i}\right)}}{2}\right)+\ln\left(\frac{1+x_{\left(z_{i}\right)}^{2}}{2}\right)-2\arctan\left(x_{\left(z_{i}\right)}\right)+0.5\pi$$

where $z_{i}$ is equal to $z_{m}$ or $z_{om}$.

(A7) $$\psi_{h\left(z_{j}\right)}=2\ln\left(\frac{1+x_{\left(z_{j}\right)}^{2}}{2}\right)$$

where $z_{j}$ is equal to $z_{T}$ or $z_{oh}$.

(A8) $$x_{\left(z\right)}={\left(1-16\frac{z}{L}\right)}^{0.25}$$

where z is equal to $z_{m}$, $z_{om}$, $z_{T}$, or $z_{oh}$.

For L > 0, it is at stable conditions:

(A9) $$\psi_{m\left(z_{i}\right)}=-5\left(\frac{z_{i}}{L}\right)$$

(A10) $$\psi_{h\left(z_{j}\right)}=-5\left(\frac{z_{j}}{L}\right)$$

Formulas (A1)–(A10) indicate that undetermined temperature endmembers are required as input in the calculation of aerodynamic resistances. At the same time, aerodynamic resistances are the required parameters for calculating the temperature endmembers. Therefore, an iterative process is executed setting the initial values of temperature endmembers as $T_{a}$ in the iteration cycle and terminating the loop when the difference between the new calculated value and the last one is less than 0.001 K or the number of iterations is greater than 500.
